# Eight Feet of Sore Throat

**Published:** 1902-10

**Authors:** 


### Eight Feet of Sore Throat.

Miss Lucy, who is in the early part of her teens, has eight feet of sore throat. Lucy is one of the giraffes at the Glen Island Zoo, New York. She caught cold one wet night during the opening week of the season, and, always a retiring and modest creature, did nothing at first to call attention to her sufferings. Before her physicians realized that anything had happened to her, she was suffering from a severe cold of the influenza type, complicated with sore throat. The water flowed from her beautiful, large, dark eyes and her pretty nose. Her vocal cords and other parts of the throat could be felt swollen and hard under the skin.

Poor Lucy developed a most excruciating cough, which was almost eternal in its slowness, owing, doubtless, to its having such a remarkable distance to travel. It was heartrending to think that every inch of that eight-foot neck was sore, stiff and inflamed. The management of Glen Island decided that something would speedily have to be done, and as a temporary relief a mild spray of one pint of cocaine was administered.

This gave Lucy marked relief, for her eyes and nose ceased to water, and she no longer showed signs of extreme pain. The thing to be done was to protect her throat during the remainder of her illness. For this purpose a band of warm, dry flannel, twenty feet long, was procured, and two men, standing on ladders, wound this carefully around poor Lucy's neck.

A sort of flannel dressing gown was then built to cover the rest of Lucy's person. An order was sent to a chemist to manufacture a nose and throat spray of appropriate size at once. This was made a similar shape to those used by human beings. The bottle part of it, however, held a full quart of liquid instead of two ounces, which the ordinary atomizer contains. The atomizing process was carried out by means of a bulb as large as a foot ball.

The mixture was made of peroxide of hydrogen. One of the keepers held this apparatus while another took the bulb and squeezed it, and Lucy, being a very amiable creature, was quickly persuaded that the treatment was for her good.

The spray was administered regularly once an hour, and at the same time a diet suitable for a giraffe with a bad cold was provided. This consisted of warm bran mash, boiled onions and hot whisky and water. Lucy liked the latter, and could not get sufficient to satisfy her. It was squeezed into her mouth from a sponge, and so anxious was she to get all that the sponge contained that it was found necessary to tie a string to it to prevent her from swallowing it. Large doses of soothing paragoric were also administered every few hours. Despite this unremitting care her temperature rose to 103 while her pulse fell to 58 a minute.

She was also suffering from an acute fever and her devoted physicians sat at her head, perched on top of ladders, all night. At daybreak, however, her watchers were delighted to see the suffering giraffe drop into a soothing sleep. When she awoke the crisis was passed. Then she made steady progress toward recovery.—*New York Journal.*

